# A QUALITATIVE ANALYSIS OF A DIGITAL FALL PREVENTION EXERCISE PROGRAM FOR OLDER ADULTS WITH INCREASED FALL RISK

**DOI:** 10.1093/geroni/igad104.3146

**Published:** 2023-12-21

**Authors:** Shannon Farrell, Nicole Bajdek, Mary Dishaw, Pamela Garabedian, Alisha Williams, Naomi Hachen, Kieran Reid, Nancy Latham

**Affiliations:** Brigham and Women’s Hospital, Boston, Massachusetts, United States; Brigham and Women’s Hospital, Boston, Massachusetts, United States; Brigham and Women’s Hospital, Boston, Massachusetts, United States; Mass General Brigham, Somerville, Massachusetts, United States; Best Buy Health, Richfield, Minnesota, United States; Best Buy Health, Richfield, Minnesota, United States; Brigham and Women’s Hospital/Harvard Medical School, Boston, Massachusetts, United States; Brigham and Women’s Hospital, Boston, Massachusetts, United States

## Abstract

Falls are a significant public health problem; one third of individuals aged 65 years or older fall each year. Strength and balance exercises reduce fall risk, but most older adults are inactive. Individuals at risk of falls need clear guidance to ensure exercises performed at home are safe and provide adequate challenge. The aim of this qualitative study was to investigate the experiences and perceptions of older adults with increased fall risk enrolled in a 3-month digitally delivered home-based fall prevention exercise program (DFP). Semi-structured interviews were conducted by an interview specialist on a sample of 16 participants (81% female, age 77.3±5.8 years). Interviews were transcribed, imported, and coded into Dedoose, a tool for qualitative analysis. Codes were refined with each interview and themes were generated from the final codes. Three themes were identified: adherence to a home-based digitally delivered fall prevention exercise program, impact of fall prevention exercises on activities of daily living (ADL), and benefits of home-balance exercises. Participants attributed adherence to the home exercise program with minimal in-person visits. Participants reported fear of falling increased as they aged; upon completion, participants felt reduced fear of falling in their ADL. Balance exercises were the most appealing due to the level of difficulty and motivation to improve balance. Participants recommended changes to improve the DFP exercise program, with majority wanting to continue the program. This qualitative analysis provides guidance to health professionals about the acceptability and recommended changes for a digitally delivered home fall prevention exercise program.

